# A chameleon-inspired stretchable electronic skin with interactive colour changing controlled by tactile sensing

**DOI:** 10.1038/ncomms9011

**Published:** 2015-08-24

**Authors:** Ho-Hsiu Chou, Amanda Nguyen, Alex Chortos, John W.F. To, Chien Lu, Jianguo Mei, Tadanori Kurosawa, Won-Gyu Bae, Jeffrey B.-H. Tok, Zhenan Bao

**Affiliations:** 1 Department of Chemical Engineering, Stanford University, Stanford, California 94305, USA; 2 Department of Electrical Engineering, Stanford University, Stanford, California 94305, USA; 3 Materials Science and Engineering, Stanford University, Stanford, California 94305, USA

## Abstract

Some animals, such as the chameleon and cephalopod, have the remarkable capability to change their skin colour. This unique characteristic has long inspired scientists to develop materials and devices to mimic such a function. However, it requires the complex integration of stretchability, colour-changing and tactile sensing. Here we show an all-solution processed chameleon-inspired stretchable electronic skin (e-skin), in which the e-skin colour can easily be controlled through varying the applied pressure along with the applied pressure duration. As such, the e-skin's colour change can also be in turn utilized to distinguish the pressure applied. The integration of the stretchable, highly tunable resistive pressure sensor and the fully stretchable organic electrochromic device enables the demonstration of a stretchable electrochromically active e-skin with tactile-sensing control. This system will have wide range applications such as interactive wearable devices, artificial prosthetics and smart robots.

## Abstract (web summary)

Some animals and insects can change the colour of their skin, but mimicking such function in man-made materials is complex. Here, the authors demonstrate an all-solution processed chameleon-inspired stretchable e-skin capable of interactive colour changes and tactile sensing.

## Graphical abstract

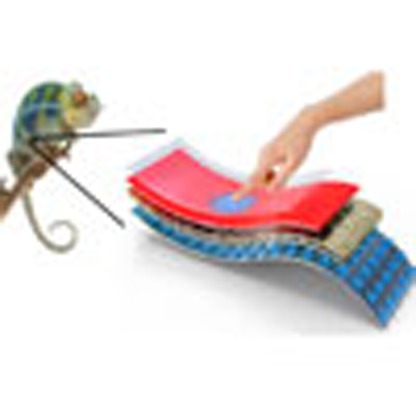

Human skin provides a remarkable network of sensors with highly sensitive pressure, temperature and vibration sensing^1^,^2^,^3^. Skin can transduce environmental stimuli into physiological signals, which are then interpreted by brain. Electronic skin (e-skin) is an artificial skin that mimics the properties of skin using electronic devices. Inspired by human skin, e-skin has been found many potential applications^4^,^5^,^6^,^7^,^8^,^9^,^10^,^11^,^12^,^13^,^14^,^15^,^16^,^17^,^18^,^19^,^20^,^21^,^22^,^23^,^24^,^25^, such as wearable devices, artificial prosthetics, health monitoring and smart robots.

Unlike human skin, both animal and insect skin exhibit additional functions, for example, the chameleon's skin has colour-changing abilities. A chameleon shifts its skin colour through controlling the skin pigment cell for purposes in camouflage^26^, temperature maintenance and communication. Since chameleons cannot generate any body heat, the colour of their skin can in turn be used to regulate their body temperature. Mimicking the colour-changing ability of chameleons can also be achieved using approaches such as mechanical or electrical control^27^,^28^,^29^,^30^,^31^. Whitesides and colleagues^31^ reported a soft machine equipped with microfluidic channels that can be colour-filled or colour-flushed by pumping a coloured liquid through the channels. Rogers and colleagues^32^ reported an adaptive optoelectronic camouflage system using a leucodye composite, which can produce black and white patterns to match its surroundings. Zhao and colleagues^33^ reported a soft material system for generating voltage-controlled on-demand fluorescent patterns that can be modulated to exhibit manifold geometries. Despite these achievements, the above devices nonetheless only demonstrated colour-changing abilities, it lacks the crucial function of skin, namely, tactile sensing. Javey and colleagues^13^ recently furthered these advancements by describing a user-interactive e-skin for instantaneous pressure visualization on a polyimide substrate. This e-skin is capable of correlating the applied pressure to the brightness of the devices as well as spatially mapping the applied pressure. However, the e-skin needs a constant bias to maintain its light/colour, which is not ideal for low power consumption system. In addition, since the device is fabricated on a plastic substrate, it is not stretchable.

Human skin is generally considered as an ‘ideal' low power consumption sensor. To mimic this property, creating a low power consumption system is highly relevant for e-skin applications. In addition, presence of both parameters in tactile sensing and stretchability are also important because tactile sensing of skin allows the body to communicate with the outside environment, whereas the stretchability of skin enables us free movement.

In the following, we describe a bio-inspired stretchable e-skin with interactive colour-changing and tactile-sensing properties (Fig. 1). This concept is realized through the development and integration of a stretchable highly tunable resistive pressure sensor (PS) and stretchable organic electrochromic devices (ECDs). This e-skin, besides detecting applied pressure, is also able to distinguish varying applied pressures through real-time visible colour change. This work demonstrates low power consumption, interactive and colour-changeable e-skin, which is readily prepared by a cost-efficient all-solution processing approach.

## Results

### Stretchable, transparent and highly tunable resistive PSs

Pressure sensing is one of the key functions of e-skin devices. Development of PSs that can mimic the pressure-sensing properties of natural skin requires proper design of both materials and devices^6^,^34^,^35^,^36^,^37^,^38^,^39^. In our previous study, we have developed a highly sensitive flexible PS with microstructured polydimethylsiloxane (PDMS) dielectric layer^6^. Various other PSs with high pressure sensitivity have been reported recently^5^,^6^,^7^,^39^,^40^. While high sensitivity is a crucial characteristic of PSs, facile implementation in real-world applications also necessitates tunable response sensitivities and tunable threshold of resistance switching range to meet different requirements. To integrate e-skin with a stretchable organic ECD, a stretchable and highly tunable resistive PS is needed. In addition, since electrochromic colour is voltage modulated, the pressure-sensitive element therefore needs to modify its voltage upon applied pressure. A comparator circuit is the most straightforward and simplest approach to implement the above requirement.

Even though many resistive PSs have been reported, their resistance value and threshold of resistance switching ranges are not easy to adjust. Furthermore, the change in resistance value is insufficient to be switched from non-conductive to highly conductive. To meet these requirements, we here developed a new type of stretchable, transparent and highly tunable resistive PS based on an elastic pyramidal-microstructured PDMS with a layer of spray-coated single-wall carbon nanotubes (SWNTs). The PS is uniquely designed to be capable of adjusting the height of the SWNTs on the pyramid, and also in controlling the resistance response and the threshold of resistance switching range.

**Figure 2:**
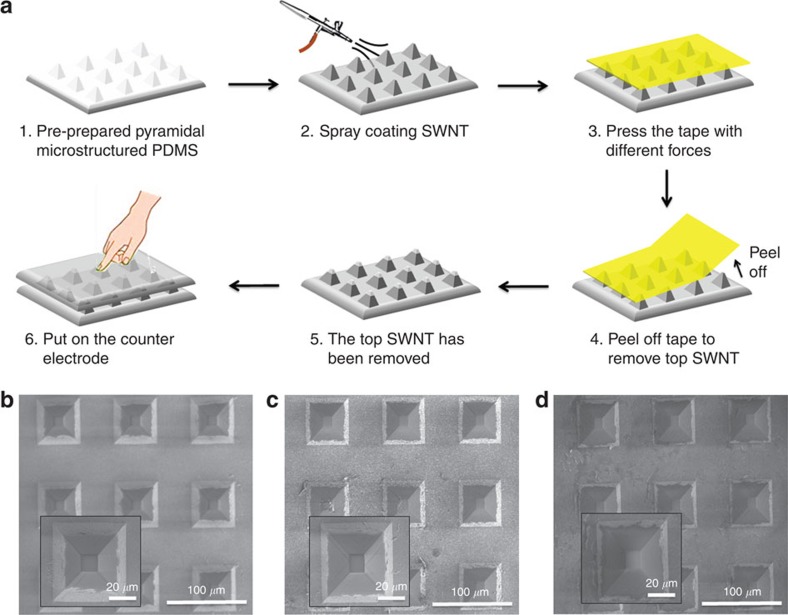
A schematic of the fabrication process and their SEM images. (**a**) A schematic for the fabrication of PSs PS-1, PS-10 and PS-30. (**b**) The SEM image of PS-1. (**c**) The SEM image of PS-10. (**d**) The SEM image of PS-30. Higher magnification SEM images of individual pyramid are provided in the inset.

The schematic process for the fabrication of the PS is shown in Fig. 2a. First, we used a pyramidal-microstructured PDMS with a 34 μm in height, 10 × 10 μm^2^ for the top, 50 × 50 μm^2^ for the base and 41 μm periodic spacing. It was fabricated using our previously described methods (Supplementary Fig. 1a)^6^,^41^. Second, the SWNT was uniformly spray coated on top of the pyramidal-microstructured PDMS surface. After the SWNT spray coating, we observed that the 10 × 10 μm^2^ top of pyramids is covered by SWNT, leading to low resistance even without any applied pressure (Supplementary Fig. 1b). Therefore, we used a kapton tape to remove the SWNT from the top of the pyramid. A key aspect of our approach is that we hypothesized that we can easily apply different pressures (that is, 1, 10 and 30 kPa) on the kapton tape to adjust the height of the SWNT on the pyramidal-microstructured PDMS. Hence, we have accordingly designated these sensors as PS-1, PS-10 and PS-30, respectively. Notably, pyramids with heights of 34 μm were used in this study because it can provide a larger dynamic range for modulating the height of SWNTs on the pyramids compared with a smaller height^6^,^17^,^42^,^43^. Indeed, it was observed that the larger the pressure applied on the tape, the less SWNT remained on the pyramids (Fig. 2b–d). This provided both the necessary tunable resistance and tunable threshold of resistance switching range for the PS. Finally, a counter electrode composed of a layer of SWNTs on a flat surface of PDMS was placed on the top of the SWNTs partially coated pyramids.

**Figure 3:**
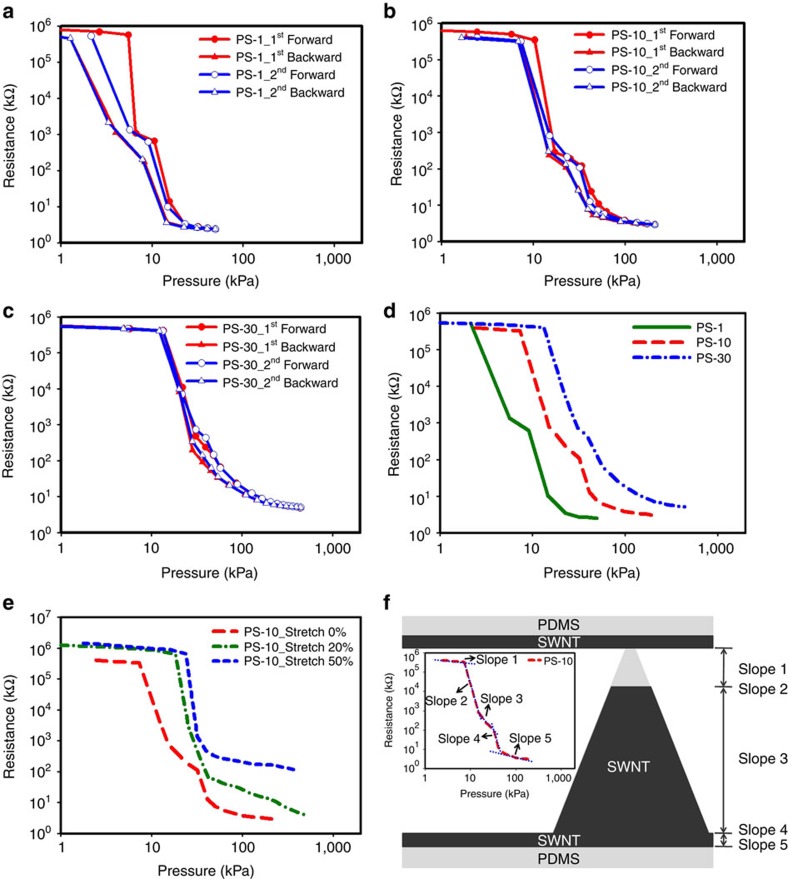
Pressure-sensing characterization of PS. The pressure response for four consecutive measurements of (**a**) PS-1, (**b**) PS-10 and (**c**) PS-30. (**d**) The comparison of the resistance response and the threshold of resistance switching range of PSs (second forward). (**e**) The comparison of the resistance response of PS-10 at different strains. (**f**) A schematic diagram of single SWNT-coated pyramidal-microstructured PDMS (inset: each slope of the resistance response of PS-10).

Figure 3a–c shows the pressure response for four consecutive measurements (that is, press, blue line; release, red line) of the pyramidal-microstructured PDMS corresponding to PS-1, PS-10 and PS-30, respectively. The resulting square pressure-sensitive pad was 1 cm^2^ in size. Slightly higher resistances were observed with PS-1 and PS-10 for the first cycle, the subsequent cycles were reproducible. Based on our design, when no pressure is applied, the sensor is non-conductive and should prevent the current flowing due to no SWNTs coating on the top of the pyramid. It becomes conductive and the resistance drops with an applied pressure. As expected, PS-1 showed the lowest resistance at the same applied pressure, which is a result of the higher coverage of SWNTs for PS-1. All the PSs PS-1, PS-10 and PS-30 exhibited changes in resistance of up to five orders of magnitude by applying pressure between 0 and 20 kPa, 0 and 50 kPa, and 0 and 165 kPa, respectively. In addition, Fig. 3d shows that the threshold of the resistance switching of PS-1, PS-10 and PS-30 was gradually increased as a function of the height of the SWNT, showing onset detection of pressures at 2.2, 7.4 and 13.3 kPa, respectively (second forward). This demonstrates that the height of the SWNTs can be related to the threshold of the resistance switching range, which is defined as the point of the resistance of the PS in which it begins to drop markedly. Our obtained results indicated that both the resistance range and the threshold of resistance switching are highly tunable through simply modifying the height of the SWNT structures. Here we target pressure range equivalent to human touch. Normal grip forces to hold objects are in the range of several kPa to several hundred kPa. The pressure applied during a handshake is in the order of several tens of kPa. Therefore, the easy and tunable design of our PS is ideal for human interactive system, which will be demonstrated in the integration section.

To test the stretchability of our sensors, we stretched PS-10 at both 20 and 50% strains. The PS still maintained its functionality, as shown in Fig. 3e. The PS-10 exhibited changes in resistance of up to five orders of magnitude at 20% strain. Even at 50% strain, it can still maintain the changes in resistance of up to four orders of magnitude. As a result, we have demonstrated stretchable and transparent resistive PSs, with the advantage of having a widely tunable dynamic range by using a simple process. To further access the resistance response, we analyse the slopes of response curve of PS. As shown in Fig. 3f, five different kinds of slopes are observed in the response curve of PS-10. As the pressure is applied, slope 1 corresponds to the counter electrode touching the non-SWNT-coated pyramid tip, but before the counter electrode touches the SWNT-coated part. During this period, we observed that the slope is nearly flat and this is because the PS is maintained in a non-conductive status. Slope 2 is when the counter electrode first comes into contact with the SWNT-coated parts of the pyramids. A sharp slope is observed in this period, since the SWNT-coated part of the pyramids provide a large sheet resistance to reduce the resistance markedly. Slope 3 is generated when the counter electrode is in contact with the SWNT-coated pyramid just before touching the base of SWNT-coated PDMS. Slope 4 is generated when the counter electrode touches the base of SWNT-coated PDMS, this is referred to as. Finally, the region where the curve of the resistance response of PS-10 becomes saturated is slope 5.

To illustrate the stability of our fabricated PS, we measured multiple cyclic data of PS-10. As shown in Supplementary Fig. 2, the resistance of the PS remained consistent at both 0 kPa (∼2 × 10^6^ kΩ) and 22 kPa (∼50 kΩ) after over 300 cycles. This observation illustrates that our resistive PS has both long-term stability and reliability as the SWNTs remained well attached on the surface of pyramidal PDMS structures. In addition, we have fabricated a 3 × 3 cm^2^ PS to demonstrate that the pyramidal structure and the SWNT removal processes for the PSs can potentially be applied to large area devices (Supplementary Fig. 3). With this PS, we observed excellent resistance response curves and reproducibility after a 10 × repeated run. Furthermore, the change in resistance is ∼5 orders of magnitude, which demonstrates that the PS can indeed be applied over a large area for practical artificial skin applications.

### Fully stretchable organic ECDs

To fabricate the stretchable interactive colour-changeable e-skin, a stretchable ECD is selected to integrate with the highly tunable resistive PS due to its ease in fabrication, multi- and tunable colours. In addition, human skin is an ideal low power consumption sensor. To mimic this property, reducing the power consumption is essential for practical e-skin applications. The ECD has the advantage of colour retention being called the ‘colour memory effect' and has also been demonstrated to be low power consuming^44^,^45^,^46^. Therefore, the development of stretchable ECD for e-skin applications is ideal to achieve both the concept of low power consumption and colour-changeable properties. In a previous report on stretchable ECD by Lee and coworkers^47^, they used an inorganic electrochromic material WO_3_ for colour switching. However, a liquid electrolyte was used that had the potential issue of liquid leakage, and will lead to a critical weakness for e-skins when exposed to a variety of wear-and-tear forces. In addition, they used a rigid platinum wire as the counter electrode, which is not stretchable, and a thick silver nanowire film is used as the non-transparent electrode, which will be difficult to implement in the multiplex ECDs and incompatible with dual-electrochromic layer. Consequently, such an ECD is not capable of stretching the counter electrode and electrolyte, and is not suitable for making the fully stretchable colour-changeable e-skin. We choose polymer-based ECD owing to their ease of colour tuning through molecular design^1^,^48^,^49^, good stretchability^8^,^50^ and promising application in printed devices^48^,^51^,^52^, thereby producing a mechanically robust and scalable ECD to overcome present obstacles. Here the stretchable polymer ECDs are designed and fabricated to be fully stretchable, including the counter electrode and electrolyte. Our ECD is fabricated on an elastic and biocompatible PDMS substrate. Thin films of SWNTs were used as transparent electrodes because of their high stretchability and good conductivity. They were coated by spray coating on the top of the PDMS substrate^4^. Various electrochromic (EC) polymers have previously reported^53^,^54^,^55^,^56^,^57^,^58^. Poly(3-hexylthiophene-2,5-diyl) (P3HT) was used as an electrochromic layer for demonstration of concept due to its good stretchability^59^,^60^,^61^,^62^. It exhibits a dark red colour in its neutral form and pale blue colour in its oxidized form.

**Figure 4:**
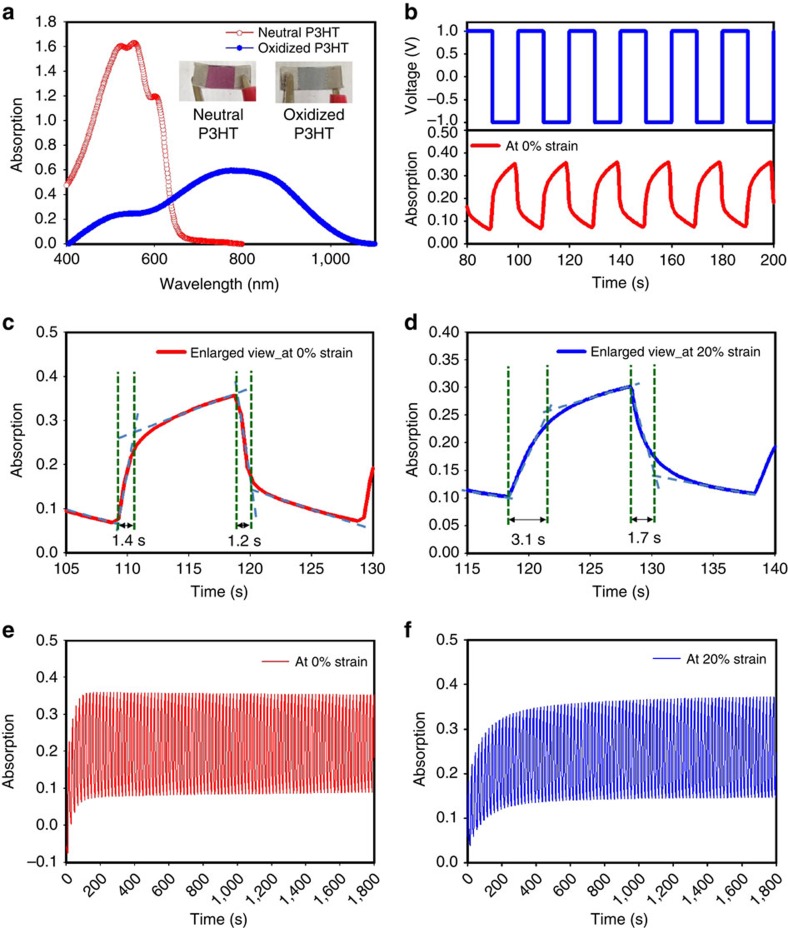
The properties of the stretchable polymer-based ECDs. (**a**) The ultraviolet-visible spectra of the neutral and oxidized P3HT. (**b**) The colour switching behaviour of the ECDs at 0% strain. (**c**) The enlarged spectra of the single switching cycle at 0% strain. (**d**) The enlarged spectra of the single switching cycle at 20% strain. (**e**) The cyclic switching at 0% strain. (**f**) The cyclic switching at 20% strain.

Figure 4a shows the ultraviolet-visible spectra of the stretchable polymer-based ECDs. The maximum absorption of the neutral P3HT was ca. 550 nm, whereas the oxidized P3HT was red-shifted to ca. 800 nm. The kinetic absorption measurement is used to determine the switching behaviour and stability of the stretchable ECDs at 550 nm through cycling the bias voltage between 1.0 and −1.0 V with a 10-s step, as shown in Fig. 4b. Notably, its low driving voltage of ±1.0 V is beneficial for the concept of low power consumption e-skin. At 0% strain, the switching time for neutral state (turning dark red) and oxidized state (turning pale blue) was 1.4 and 1.2 s, respectively (Fig. 4c). At 20% strain, the switching time for neutral state (turning dark red) and oxidized state (turning pale blue) was only slightly increased at 3.1 and 1.7 s, respectively (Fig. 4d). Both the stretchable organic ECDs at both 0 and 20% strains showed stable cyclic switching, and the colour contrast was maintained at over 90% even after long-term measurement (Fig. 4e,f). In addition, the ECDs at 50% strain showed the function of the switching cycle (Supplementary Fig. 4). However, the switching speed was degraded because of the increased resistance. Notably, we chose to use commercially available P3HT to demonstrate the fully stretchable ECDs. We believe that improvements of device performance can be further achieved by design and synthesis of improved electrochromic polymers.

**Figure 5:**
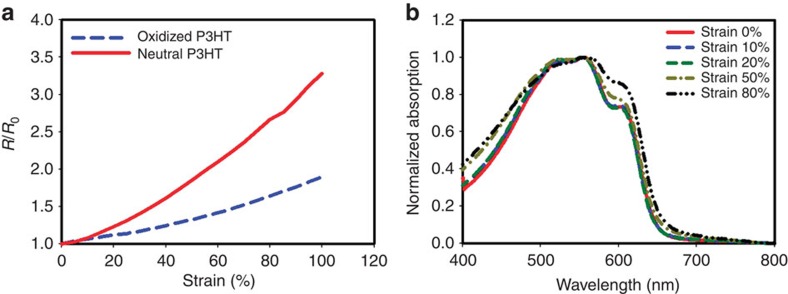
Mechanical and absorption characteraization of ECDs. (**a**) The resistance comparsion of neutral and oxidized P3HT for strains ranging from 0 to 100%. (**b**) The absorption spectra of P3HT ECD at different strains.

To elucidate the relationship between strain and electrical properties of the neutral and oxidized P3HT/SWNT stack in ECDs, we measured the resistance versus strain for both the neutral and oxidized P3HT/SWNT films. The films were first both soaked in the liquid electrolyte (0.1 M LiClO_4_ in CH_3_CN) for 30 s before the stretching measurement. Figure 5a plots the normalized resistance of these two thin films when stretched from 0 to 100% strain. The resistance of the oxidized P3HT at 100% strain increased only ∼1.9 times, whereas the neutral P3HT was increased to ∼3.2 times at 100% strain. We can observe that at 20% strain, the resistance of the oxidized and neutral P3HT was only increased 1.23 and 1.12 times compared to its original resistance, respectively. If we stretch the thin films to 50% strain, the resistance of the neutral and oxidized P3HT was increased to 1.85 and 1.33 times, respectively. We speculated that the much higher increase in the resistance of neutral P3HT led to the degradation of the switching speed of ECD at 50% stain. The slower resistance increases in the oxidized P3HT thin film may be because of the higher concentrations of holes in the oxidized P3HT thin film, helping to maintain more conductive pathways even at elevated strains to inhibit the resistance increase. In addition, to further investigate the effects of strain on the ECDs, we compared the absorption spectra of P3HT under different strain levels (Fig. 5b). The absorption shoulder of P3HT at 600 nm, typically assigned to the aggregation state of P3HT, increased when subjected to >50% strain. This result suggests that the fraction of aggregated polymer increased during stretching, most likely due to strain-induced chain extension and subsequent crystallization.

A key parameter for wearable electronics is its weight. To demonstrate the lightweight capability, we used an ultrathin 1.2-μm-thick polyethylene naphthalate (PEN) film to fabricate the organic ECDs. In addition, the ultrathin PEN or polyethylene terephthalate has exhibited the capability of geometric stretchability^11^. The fabrication process is similar to that of organic ECDs on PDMS substrate. The device weighed only 9.3 mg cm^−2^, which is lighter than the weight of a similarly sized device on textile, such as a conventional labcoat made from cotton polyester blend (16.7 mg cm^−2^; Supplementary Fig. 5). These results indicate that organic ECDs are potentially promising for wearable devices. As a result, we have demonstrated that our stretchable organic ECDs have fast colour responses, are transparent and lightweight.

### Chameleon-inspired stretchable e-skin integration

**Figure 6:**
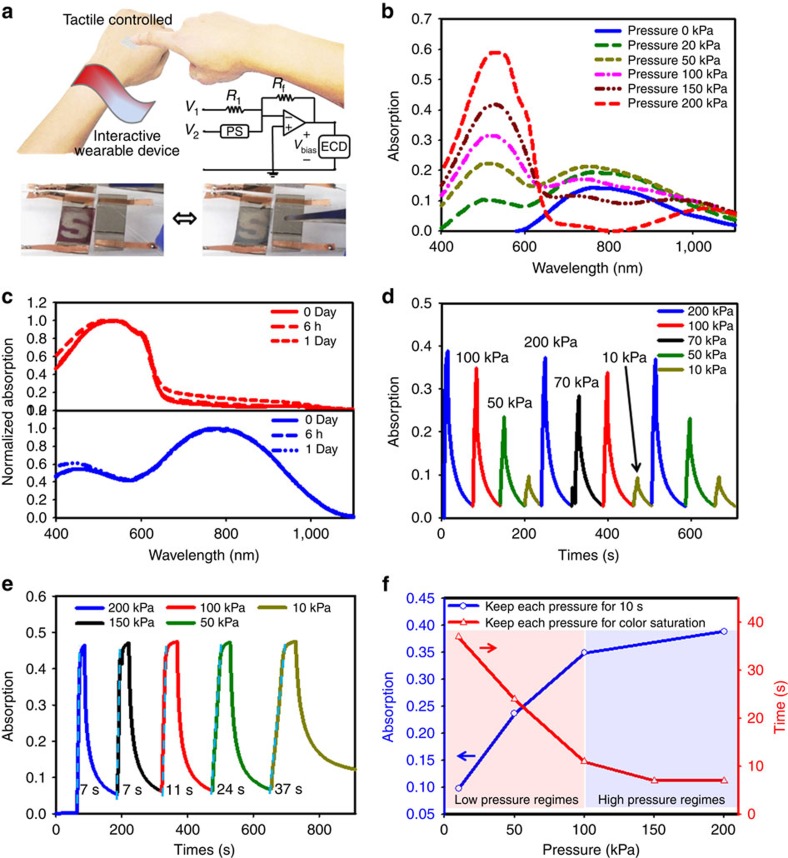
The schematic layout and measurement of the integrated systems. (**a**) The schematic layout of interactive colour-changeable e-skin, the layout of the circuit and the photos of PSEC. (**b**) The absorption spectra related to the pressures. (**c**) The colour retention properties of ECDs. (**d**) The real-time absorption changes corresponding to the various applied pressures, in which each pressure was mainatined for 10 s. (**e**) The speed of colour saturation for different pressures, each pressure was applied till colour saturation for all cases. (**f**) The absorption and time versus the pressure for both the low- and high-pressure regimes.

To produce a prototype, an integrated stretchable PS and stretchable ECD, denoted as ‘PSEC', is fabricated. The schematic layout of the integrated system and its schematic circuitry is shown in Fig. 6a. Based on this integration, we can control the colour by simply applying various pressure levels. Figure 6b shows the spectroscopic change measured by ultraviolet-visible absorption spectrum by applying different pressures from 0 to 200 kPa. Each of the applied pressure was first maintained for 10 s, and the device was then turned off and the absorption spectrum was subsequently measured. We observed an absorption peak at 800 nm for the oxidized P3HT before any pressure was applied. With increasing pressure, we observed the appearance of another absorption peak at 550 nm (which we attribute it due to the formation of neutral P3HT), and its intensity was observed to increase with increasing applied pressure. Various absorption spectrum/colours were used to further quantify the corresponding magnitude of the applied pressure. Through introducing various colours of the electrochromic polymers, the PSEC system could potentially be designed with a wide range of colours and, more importantly, be modulated by various pressures. As such, we will then be able to distinguish the different applied pressure level via the skin colours. In addition, to realize the power consumption property of the PSEC system, we measured if the absorption band/colour of the ECD at either natural or oxidized states. As Fig. 6c shown, both the absorption band/colour of neutral and oxidized P3HT can be maintained for 6 h at ambient environment without applying any additional bias. We only observed a slight increase in the absorption band of the ECDs after 1 day. This demonstrates that our colour-changeable e-skin does not require a constant bias to maintain the colour, which is important for the concept of a low power consumption e-skin. We also note that commercial electrochromic polymer P3HT was used to produce the prototype. Hence, further improvement can be achieved by using the modified electrochromic polymers or better encapsulated processing.

We further investigated the response time of colour changes as a function of applied pressure using ultraviolet-visible measurements. We applied various pressures to the PSEC and maintained each pressure for 10 s. Figure 6d shows that the largest changing response of absorption was obtained using the highest pressure of 200 kPa. We then decrease the applied pressure down to 150, 100, 70, 50 and 10 kPa, and the corresponding changes in absorption were observed. We observed that the various applied pressures correspond to different absorption spectrum/colour. Thus, the real-time absorption change can be used to determine the magnitude of the pressure that was applied. In addition, various pressures were applied to the PSEC system until the colour of the ECDs was saturated (Fig. 6e). The time required to reach colour saturation was found to decrease upon increasing applied pressure. For example, the applied pressure of 200 kPa required 7 s to achieve colour saturation, whereas an applied pressure of 10 kPa can only reach colour saturation after 37 s. Notably, the value of the slope can be used to quantify the magnitude of pressure we applied. The PSEC system took a longer time to achieve colour saturation compared with our stretchable polymer-based ECDs described in this work. We propose that the slower response is because the pressure-driven voltage has a time delay to reach the proper voltage. The slope of each response curve was decreased when a lower pressure was applied. Importantly, based on the results of the response–time measurement, the lighter pressure regime from 0 to 100 kPa (red region) provides a marked absorption change and saturation time change (Fig. 6f), whereas the higher pressure regime from 100 to 200 kPa (blue region) shows only a slight absorption change and saturation time change. Consequently, the PSEC system can be operated at high pressures to achieve quicker colour saturation. Alternatively, it can be driven at a lower pressure to provide a larger dynamic range to fine-tune the various colours.

**Figure 7:**
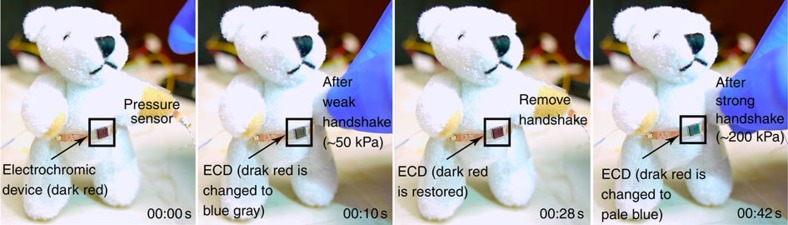
An interactive colour-changing and tactile-sensing e-skin. Sequential images of a teddy bear show the expression of tactile sensing into visible colour changes. In specific, the original colour of the ECD is changed from dark red to blue grey upon a weak applied pressure (∼50 kPa), reverts back to dark red upon pressure release and changes to pale blue upon a strong applied pressure (∼200 kPa; left to right images). The number at the bottom right corner of each image indicates the elapsed time.

As a demonstration of an e-skin with both interactive colour-changing and tactile-sensing properties, a stretchable PS and a stretchable colour-changeability are mounted and connected onto the abdomen and the back of hand of a commercially purchased teddy bear (Fig. 7). Upon applying a weak handshake (∼50 kPa), the colour of ECD turned from dark red to blue grey. Releasing the handshake reverts the colour to dark red, whereas applying a strong handshake (∼200 kPa) changes the colour again to pale blue. This demonstrates that the feasibility of expressing information from tactile sensing into visible colour changes, and also the tunability of the skin colour related to the various pressures we applied, providing an important function of certain animal skins (Fig. 7 and Supplementary Movie 1). Notably, for the user-interactive devices, the toxicity and carcinogenicity of carbon nanotubes have raised concerns as they have rather similar shapes as asbestos. Previous reports have demonstrated that the longer and thicker carbon nanotubes (lengths >5 μm and diameter >20 nm) will induce significantly more DNA damage and inflammation compared with the lower aspect ratio^63^,^64^. Here we use the much shorter and smaller diameter SWNT (bundle lengths range from 0.5 to 1.5 μm, along with an average bundled diameters of 4–5 nm), which should greatly reduce the potentially adverse effect. Furthermore, we also consider that proper encapsulation of this system is needed. A number of elastic substrates, such as silicone, polyurethane or fluoroelastomers, are biocompatible and highly stretchable^65^,^66^,^67^. They are also easily processed. Therefore, we believe that encapsulation with such elastomers is a potentially compliant method for further e-skin applications.

## Discussion

In summary, we have successfully demonstrated a chameleon-inspired stretchable e-skin capable of interactive colour changes and tactile-sensing properties. This was accomplished via a stretchable, transparent and highly tunable resistive PS. In addition, fully stretchable organic ECDs were fabricated and demonstrated by all-solution processing. Our fabricated PS was observed to demonstrate a wide resistance value range, and, more importantly, the threshold of resistance switching range is tunable by modifying the height of SWNTs on microstructural pyramids. Integration with e-skin rendered this fully stretchable organic ECD electrochromically active. A stretchable colour-changeable e-skin with tactile-sensing control was also demonstrated, in which the applied pressure on the e-skin can be directly expressed through colour changes. Last, we demonstrated that this stretchable colour-changeable e-skin can maintain its skin colour without any applied pressure, indicating its low power consumption. In our future studies, we aim to introduce various colours of electrochromic polymers and array designs to enable a wider and more dynamic colour range for high contrast and high resolution. Such systems should be promising for applications in interactive wearable devices, military applications, artificial prosthetics and smart robots.

## Methods

### Characterization

The absorption measurement was carried out by Agilent Cary 6000i UV/Vis/NIR. Scanning electron microscopy (SEM) was performed using an FEI Magellan 400 XHR microscope with a 5-kV accelerating voltage and 25 pA current. The conductivity of the SWNT/PDMS substrate was measured using a standard four-point probe method at room temperature. The resistance was obtained using an Agilent E4980A Precision LCR meter. The stretch property was obtained using the home-made stretchable station. PS cycling measurements were performed on a mechanized *z* axis stage (Newmark Systems, 0.1 mm resolution), and a force gauge (Mark 10) was used to apply loads to the 1-cm^2^ pressure-sensitive pad on a custom-built probe station. The load values were recorded by a precision balance.

### Preparation of PDMS substrate

A 10:1 mixture of PDMS elastomer base (Sylgard 184, Dow Corning) to curing agent was mixed and stirred for 2 min. The mixture was then evacuated in a vacuum desiccator until the air bubbles were no longer visible. Thereafter, the PDMS mixture was poured into a Petri dish (diameter=150 mm). Finally, the Petri dish containing the PDMS mixture was placed on a hotplate for 8 h at 60 °C to cure the PDMS. It is important to have a leveled surface on the hotplate to ensure the PDMS thickness uniformity.

### Preparation of pyramidal-microstructured PDMS

A patterned silicon mould (with 34 μm height, 10 × 10 μm^2^ top, 50 × 50 μm^2^ base and 41 μm periodic spacing pyramidal microstructure) was prepared by fluorination treatment after oxygen plasma for 5 min at 200 W. A 10:1 mixture of PDMS elastomer base (Sylgard 184, Dow Corning) to curing agent was mixed and for 2 min and was then evacuated in a vacuumed desiccator until the air bubbles were no longer visible. The PDMS mixture was then poured onto the patterned silicon mould. Finally, the PDMS-covered silicon mould was placed on a hotplate for 8 h at 60 °C to cure the PDMS.

### Preparation of SWNT solution

Arc-discharge SWNT (2.5 mg), purchased from Carbon Solution, Inc (P2-SWNT) was dispersed in NMP (*N*-methylpyrrolidinone) (17 ml) by sonicating the solution for 30 min in an ultrasonicator (Cole Parmer) at 30% amplitude. Subsequently, the solution was centrifuged at 8,000 r.p.m. for 30 min, and the top 80% supernatant was extracted out for spray coating.

### Spray coating of SWNT

The PDMS substrate and pyramidal-microstructured PDMS substrate were first activated with ozone for 20 min and were then placed on a hotplate at 200 °C. The SWNTs were uniformly spray coated on top of the PDMS surfaces at a distance of ∼15 cm by using an airbrush pressure of 60 p.s.i. until the desired resistance was reached (∼2.0 kΩ Sq^−1^).

### Fabrication of stretchable and ultrathin ECDs

The SWNT/PDMS stack was created by spin coating the P3HT (10 mg ml^−1^ in chloroform) onto the SWNTs at 1,000 r.p.m. for 1 min to form a polymer thin film. Thereafter, the gel electrolyte (LiClO_4_:poly(methyl methacrylate):PC:CH_3_CN=3:7:20:70 by weight) was brush coated on top^68^. Finally, the other SWNT-coated PDMS counter electrode was placed on the P3HT-coated SWNT/PDMS substrate to assemble the ECDs. For the ultrathin ECDs, the 1.2-μm PEN (Tenji Dupont Film, TeonexFilm Q70) was well attached on a PDMS substrate. The fabrication process of this ultrathin PEN substrate is similar to that of PDMS. The difference between the processing of these two substrates is that we spray-coated SWNT at a lower temperature of 140 °C on the PEN substrate due to the glass transition temperature of PEN is 155 °C. We used a laser-cut PDMS membrane that had S-shape as a stencil mask to make the patterned ECDs.

### Fabrication of resistive PSs

The pyramidal-microstructured PDMS (34 μm height, 10 × 10 μm^2^ top, 50 × 50 μm^2^ base and 41 μm periodic spacing) was fabricated using the same method as reported previously and described above^6^,^32^. A layer of SWNTs was then uniformly spray coated on top of the pyramidal-microstructured PDMS surface. A kapton tape was applied with different pressures (that is, 1, 10 and 30 kPa) to remove the SWNT from the pyramidal surface, designated as PS-1, PS-10 and PS-30, respectively. Finally, another SWNT/PDMS was used as the counter electrode and was placed on the top of SWNT-coated pyramidal-microstructured PDMS to give the tunable resistive PS.

### PSEC system integration

The PSEC system is fabricated by integrating the stretchable ECD and resistive PS into a summing amplifier circuit. As depicted in Fig. 6a, the summing amplifier applies a bias voltage (*V*_bias_) across the ECDs as a function of the PS resistance (*R*_ps_). This behaviour is governed by the following equation:


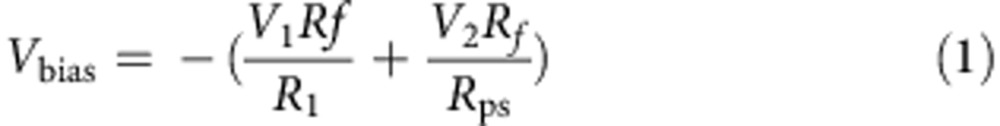


As determined by the bias minimums for switching between neutral and oxidized states *V*_1_ and *V*_2_ were, respectively, set to −1 and 1 V. Both *R*_1_ and *R*_f_ are diodes with a resistance of 30 kΩ, and *R*_f_ a diode resistance of 30 kΩ. *R*_ps_ is the resistance of the PS. Without pressure, *R*_ps_ is several orders of magnitude larger than *R*_f_, resulting in a bias voltage of −1 V. As pressure is applied, *R*_ps_ decreases, and *V*_bias_ approaches +1 V.

### Measurement of the absorption spectrum versus applied pressure

The PSEC system was integrated with the stretchable ECD and resistive PS as described above. To characterize the impact of applied pressure on the real-time absorption change, the ECD was attached on the thin-film stage of Agilent Cary 6000i UV/Vis/NIR. As different pressures were applied on the PS, the colour change of the ECD can be measured by the Agilent Cary 6000i UV/Vis/NIR, and the applied pressure on the PS was determined by a balance simultaneously.

## Additional information

**How to cite this article:** Chou, H.-H. *et al.* A chameleon-inspired stretchable electronic skin with interactive colour changing controlled by tactile sensing. *Nat. Commun.* 6:8011 doi: 10.1038/ncomms9011 (2015).

## Supplementary Material

Supplementary Figures Supplementary Figures 1-5

Supplementary Movie 1 A movie of a teddy bear shows the expression of tactile sensing into visible colour changes.

## Figures and Tables

**Figure 1:**
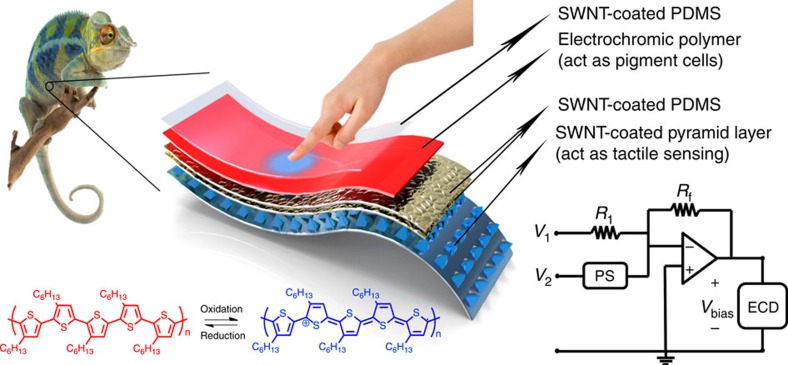
Illustration of the concept of a chameleon-inspired e-skin. Also shown are (bottom left) the structures of both the neutral and oxidized states of the electrochromic polymer in poly(3-hexylthiophene-2,5-diyl, P3HT), and (bottom right) a schematic of the circuit layout (PS, pressure sensor; ECD, electrochromic device). (Ambanja panther chameleon and young hand images from www.123rf.com).
