# Supplementary material for: A chameleon-inspired stretchable electronic skin with interactive colour changing controlled by tactile sensing
Source: Nat Commun. 2015 Aug 24;6:8011. doi: 10.1038/ncomms9011 (PMC4560774; doi:10.1038/ncomms9011)
Supplement: Supplementary Figures — 1-5 [file ncomms9011-s1.pdf]

Supplementary Information

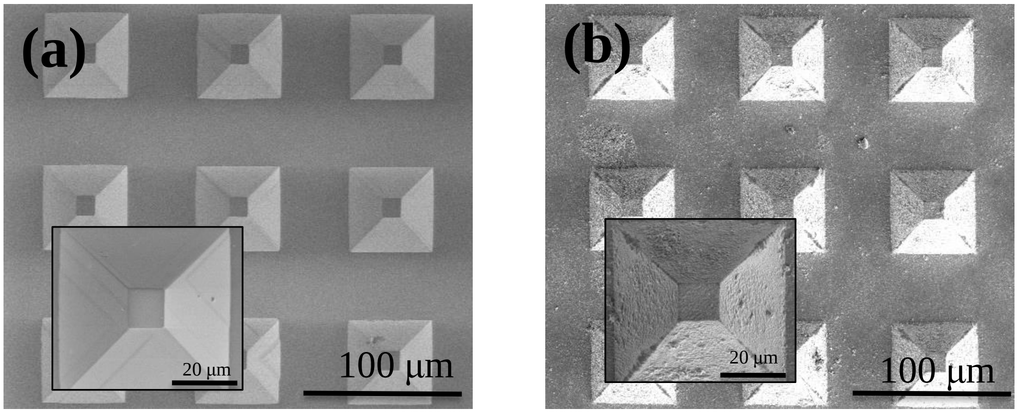

Supplementary Figure 1. The SEM images of the pressure sensor. (a) The SEM image of the pre-prepared pyramidal microstructured PDMS substrate. (b) The SEM image of the SWNT spray-coated pyramidal microstructured PDMS substrate.

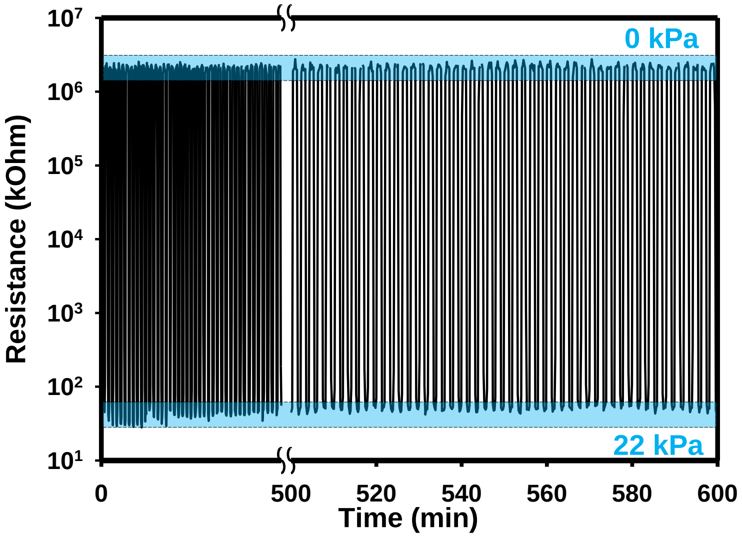

Supplementary Figure 2. The multiple cyclic data of PS-10. The resistance of the pressure sensor remained consistent at both 0 kPa (~2×10^6 kOhm) and 22 kPa (~50 kOhm) after over 300 cycles. Each cycle takes about 2 minitues.

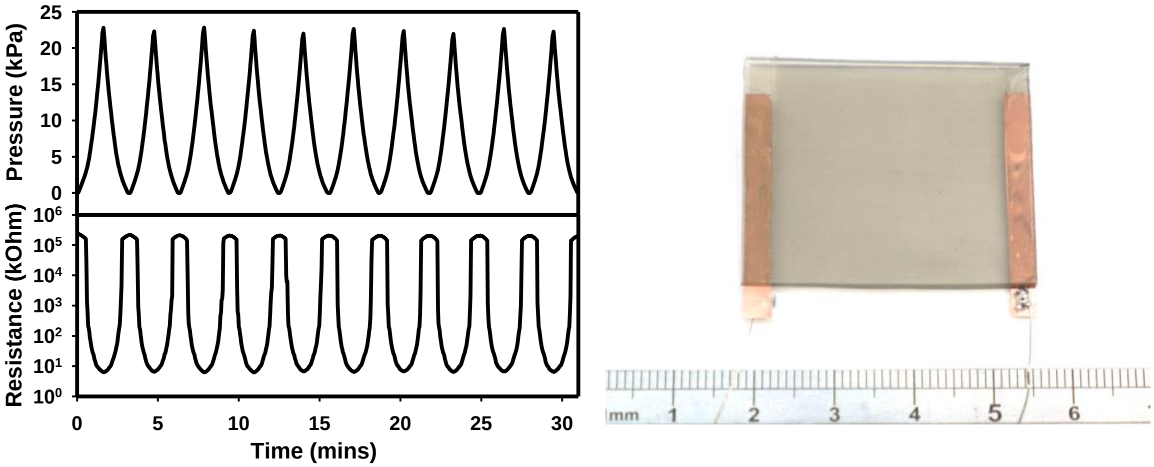

Supplementary Figure 3. The characteristic and photo of the 3 × 3 cm^2 pressure sensor. The resistance response of the large area pressure sensor and its photo with proportional scale.

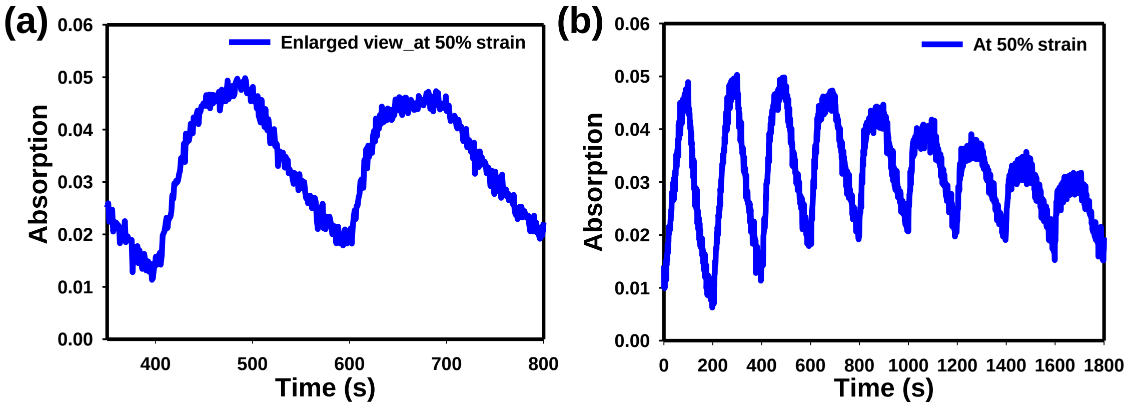

Supplementary Figure 4. The properties of the tranpsranet and stretchable polymer-based electrochromic devices. (a) The enlarged spectra of a single switching cycle at 50% strain. (b) The cyclic switching at 50% strain.

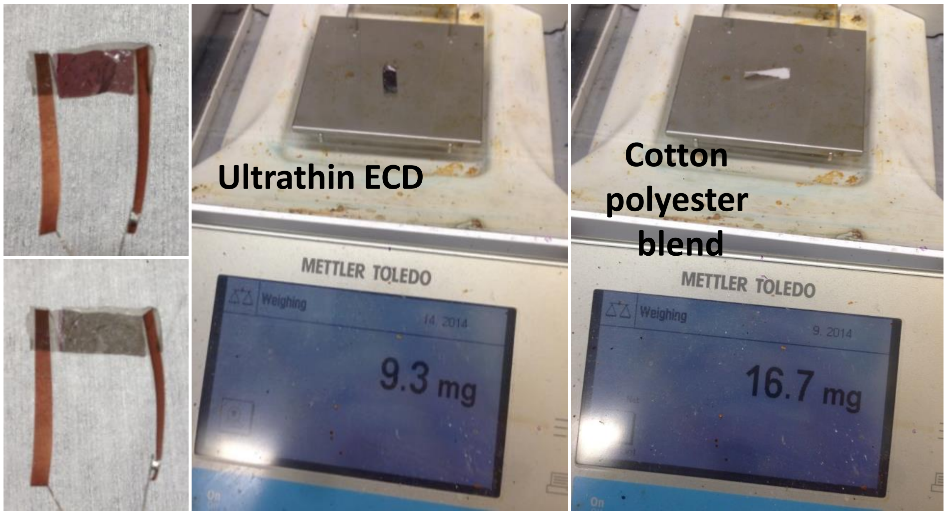

Supplementary Figure 5. The ultrathin ECDs and the weight of the ECD and a piece of cotton polyester. The size of the conventional textile is similar to that of ultrathin ECDs.
